# Correction: ‘Early to Mid-Holocene human activity exerted gradual influences on Amazonian forest vegetation’ (2022), by Nascimento *et al.*

**DOI:** 10.1098/rstb.2023.0328

**Published:** 2023-12-04

**Authors:** Majoi N. Nascimento, Britte M. Heijink, Mark B. Bush, William D. Gosling, Crystal N. H. McMichael

**Keywords:** prehistoric human impacts, cultural landscape, vegetation change, pollen, charcoal, palaeoecological synthesis

*Phil. Trans. R. Soc. B* **377**, 20200498 (Published online 7 March 2022). (https://doi.org/10.1098/rstb.2020.0498)

An incorrect version of figure 3 appeared in the published article. Symbols indicating data that are essential to the conclusions of the manuscript were not shown correctly.

**Figure 3.**
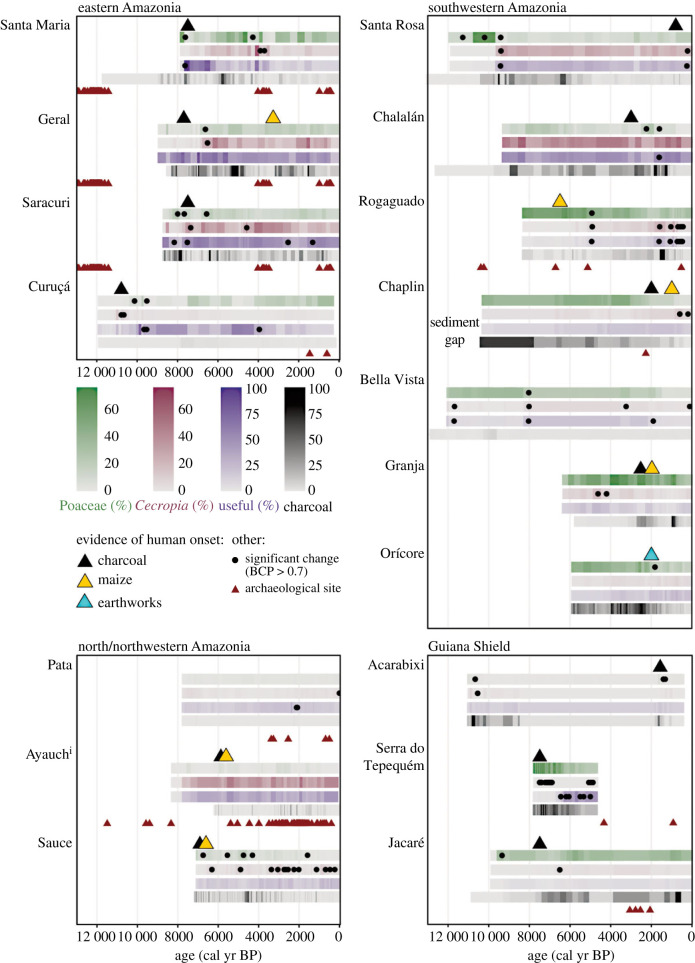
Abundances of Poaceae, Cecropia, useful species and charcoal, and the timing of the detected significant changes in vegetation per metric using a BCP analysis, compared with evidence for the onset of human activity around lakes (based on the interpretation of the original author(s)). The timing of dated archaeological material located within a 100 km radius of each lake is shown in relation to the palaeoecological data. Since the presence of humans within 100 km radius (from archaeological sites) does not guarantee that humans were using the area around the lake, vegetation changes are compared with the timing of the human onset determined by the author(s) of the original papers. The timing of archaeological occurrences within 100 km radius of the lake is used to evaluate if the pattern of occupation is synchronous between the two types of evidence and if the vegetation histories observed in the lake records cover the possible first regional human impact. The full list of palaeoecological lake records is available in electronic supplementary material, S2. (Online version in colour.)

The corrected figure is shown below and has also been corrected on the publiser's website.

